# Network analysis on psychopathological symptoms, psychological measures, quality of life and COVID-19 related factors in Chinese psychiatric patients in Hong Kong

**DOI:** 10.1186/s12888-024-05690-7

**Published:** 2024-04-12

**Authors:** Vivian Shi Cheng Fung, Joe Kwun Nam Chan, Eileena Mo Ching Chui, Corine Sau Man Wong, Ryan Sai Ting Chu, Yuen Kiu So, Jacob Man Tik Chan, Albert Kar Kin Chung, Krystal Chi Kei Lee, Heidi Ka Ying Lo, Calvin Pak Wing Cheng, Chi Wing Law, Wai Chi Chan, Wing Chung Chang

**Affiliations:** 1 Department of Psychiatry, School of Clinical medicine, LKS Faculty of Medicine, the University of Hong Kong, Pok Fu Lam, Hong Kong; 2 Department of Psychiatry, Queen Mary Hospital, Hospital Authority, Kowloon, Hong Kong; 3 School of Public Health, LKS Faculty of Medicine, the University of Hong Kong, Pok Fu Lam, Hong Kong; 4 State Key Laboratory of Brain and Cognitive Science, the University of Hong Kong, Pok Fu Lam, Hong Kong; 5 Department of Psychiatry, The University of Hong Kong Queen Mary Hospital, Pokfulam, Hong Kong

**Keywords:** Network analysis, COVID-19, Psychiatric patients, Psychopathology, Anxiety, Mental disorders

## Abstract

**Background:**

Psychiatric patients are susceptible to adverse mental health impacts during COVID-19, but complex interplays between psychopathology and pandemic-related variables remain elusive. This study aimed to investigate concomitant associations between psychopathological symptoms, psychological measures and COVID-19 related variables in Chinese psychiatric patients during the peak of fifth pandemic wave in Hong Kong.

**Methods:**

We employed network analysis to investigate inter-relationships among psychopathological symptoms (including depression, anxiety, post-traumatic stress disorder-like [PTSD-like] symptoms, insomnia, psychotic symptoms), cognitive complaints, health-related quality of life, loneliness, resilience and selected pandemic-related factors in 415 psychiatric outpatients between 28 March and 8 April, 2022. Network comparisons between genders, diagnosis (common mental disorders [CMD] vs. severe mental disorders [SMD]), and history of contracting COVID-19 at fifth wave were performed as exploratory analyses.

**Results:**

Our results showed that anxiety represented the most central node in the network, as indicated by its highest node strength and expected influence, followed by depression and quality of life. Three comparatively strong connections between COVID-19 and psychopathological variables were observed including: fear of contagion and PTSD-like symptoms, COVID-19 stressor burden and PTSD-like symptoms, and COVID-19 stressor burden and insomnia. Network comparison tests revealed significant network structural difference between participants with history of contracting COVID-19 and those without, but showed no significant difference between genders as well as between CMD and SMD patients.

**Conclusions:**

Our findings suggest the pivotal role of anxiety in psychopathology network of psychiatric patients amidst COVID-19. Pandemic-related variables are critically associated with trauma/stress and insomnia symptoms. Future research is required to elucidate potential network structural changes between pandemic and post-COVID periods.

**Supplementary Information:**

The online version contains supplementary material available at 10.1186/s12888-024-05690-7.

## Introduction

Literature has consistently shown the adverse effect of COVID-19 on individuals’ physical and mental health. Despite imposing public-health policies to contain the spread of COVID-19, the emergence of the Omicron-variant had led to the fifth pandemic wave in Hong Kong (HK) in 2022. It was the most severe outbreak of COVID-19 in HK, resulting in an estimated 60% of the population (approximately 4.4 million) having contracted the infection, with 3.73 deaths per thousand, the highest death rate worldwide at the time [1, 2]. People with pre-existing mental illness constitutes a vulnerable population during the pandemic, as they have higher risk of COVID-19 infection [3, 4] and related mortality [5], and experienced more pronounced psychological distress relative to the general population [6–8]. Notably, the complex interplay between psychopathological symptoms and pandemic-related variables among psychiatric patients are understudied and remain to be clarified. In this study, we employed network analysis to address this important question in the context of the fifth wave of COVID-19 in HK.

Network analysis approach conceptualizes psychopathology as a result of a complex, dynamic system of interconnections between symptoms [9], as opposed to previous notion of underlying common latent cause [10]. This method allows visualization of associations between variables in the form of a network while estimating relative importance of variables within the network. The resultant information not only facilitates identification of important variables in contributing to the genesis of psychopathology, but also variables that bridge between different psychopathologies [9]. In fact, network analysis has been increasingly applied to examine psychopathological network in the general population during COVID-19. For instance, a recent network-analytic study found that anxiety/fear about COVID-19 linked significantly with depression and anxiety symptoms [11]. Another report revealed depression as the most central node in the network during a lockdown period [12]. Relatively fewer network-analytic research has been conducted on psychiatric patients during the pandemic, and accumulating data have demonstrated strong connections between depression and anxiety symptoms [13, 14]. Longitudinal data and more recent cross-sectional research using data in the pandemic showed that anxiety had emerged as the core psychopathological symptom during the pandemic [15–17], and anxiety and COVID-19 related worries were significantly associated with other symptoms such as suicidal ideation in the network of clinical psychopathology [16, 18]. Nonetheless, these studies primarily focused on the associations among psychopathological items within one or two symptom scales, without taking into consideration potential influence of COVID-19 specific factors such as fear of contagion, pandemic-related stressors, distress due to social-distancing measures, to name a few. There is a paucity of network-analytic studies evaluating relationships across a more comprehensive array of psychopathological variables among individuals with mental disorders.

To this end, the current study employed a network analysis approach to investigate complex inter-relationships between psychopathological symptoms, psychological measures of resilience and loneliness, cognitive complaints, health-related quality-of-life, and COVID-19 related factors in a representative sample of Chinese psychiatric outpatients during the peak of fifth pandemic wave in Hong Kong (HK). Specifically, we aimed to: (1) examine the associations among the aforementioned array of variables, particularly those linked to COVID-19 related factors; (2) identify the most central variable in the network and clarify its associations with other variables; and (3) compare network difference between genders, diagnosis (common vs. severe mental disorders) and history of contracting COVID-19 infection.

## Methods

### Participants and setting

A total of 415 Chinese psychiatric patients aged 18–64 years were recruited from psychiatric outpatient clinics in HK West Cluster, a catchment area with a population of approximately 550,000, between 28 March and 8 April, 2022 (i.e., during the peak of the fifth COVID-19 wave in HK). Patients with learning disabilities, history of head trauma or neurological disease, or were unable to read Chinese language were excluded. Psychiatric diagnosis was based on ICD10 criteria and was ascertained by reviewing medical-records in public psychiatric services. Participants were further categorized into those with common-mental-disorders (CMD, including depressive and anxiety disorders) and severe-mental-disorders (SMD, including schizophrenia-spectrum disorders and bipolar disorder). The study was performed in accordance with the Declaration of Helsinki, and was approved by the local institutional review board (UW 22–202). Written informed consent was obtained from all participants before study assessment.

### Study assessment

Self-rated questionnaire was administered in this cross-sectional study. Psychopathological symptom assessment included the following: Depressive and anxiety symptom severity were measured by Patient Health Questionnaire-9 (PHQ-9) [19] and Generalized Anxiety Disorder-7 scale (GAD-7) [20], respectively, with both scales using a 4-point Likert scale ranging from 0 (never) to 3 (nearly every day). Total score for depression ranged 0–27, whereas that for anxiety ranged 0–21. A modified version of Impact of Event Scale-Revised (IES-R) [21] specific to COVID-19 was administered to measure PTSD-like symptoms on a 5-point Likert scale (0 [never] to 4 [always]), with total score ranged 0–24. Insomnia symptoms were assessed using Insomnia Severity Index (ISI) [22]. Positive symptom subdomain items (4 items) of 15-item Community Assessment of Psychic Experiences Scale–Chinese version (CAPE-C15) [23] was employed to assess positive psychotic symptoms. Patients rated their symptom frequency on a 4-point Likert scale (1 [never] to 4 [nearly always]). For all symptom scales, higher scores indicated greater symptom severity. Cognitive impairment was measured by a self-report questionnaire, adapted from Cognitive Complaints in Bipolar Disorder Rating Assessment (COBRA) [24], which has been applied in a recent study on psychiatric patients during COVID-19 [25]. This adapted questionnaire comprised 5 items reflecting cognitive complaints manifested in everyday scenario including attention, processing speed, memory, learning and executive function (rated on frequency of cognitive complaints on a 4-point Likert scale, ranging from 0 [never] to 3 [nearly every day]) [25]. Health-related quality-of-life was assessed using the 8-item Short-Form Health Survey (SF-8) [26], with higher scores indicating better quality-of-life (item 1 score was reversed). We measured two psychological measures, namely loneliness and resilience. Loneliness was assessed by the UCLA 3-item Loneliness Scale [27] on a 3-point Likert scale (1 [hardly ever] to 3 [often]), with higher scores indicating greater loneliness. Resilience was assessed using the Brief Resilience Scale (BRS) [28] on a 5-point Likert scale (1 [strongly disagree] to 5 [strongly agree]), with higher scores indicating greater resilience. Selected COVID-19 related factors were evaluated, comprising history of contracting COVID-19 during fifth wave, fear of contagion, number of pandemic-related stressors and distress due to social-distancing measures. Details of COVID-19 related factors are summarized in Table 1.

**Table 1:** Description of questions designed for each COVID-19 related factors measured

Variables	Description
5th wave infection	Participants were first asked whether they had contracted COVID-19 before. If participants chose “yes”, they were asked if the latest infection was in “Dec 2021 or before” or “Jan 2022” or after (during fifth wave of COVID-19 outbreak).
Fear of contagion	Participants were asked to what extent they were fear of contracting COVID-19 using a 11-point Likert scale (0 = Not afraid at all, 10 = Extremely afraid).
Number of COVID-19 stressors experienced	Participants were asked the amount of stress they experienced during fifth wave in each of the listed aspects respectively. Eight aspects were assessed in total using a 5-point Likert scale ranging from 0 (not stressed) to 4 (extremely stressed): (1) financial, (2) work, (3) physical health, (4) mental health, (5) food and supplies, (6) medicine, (7) family relationship, (8) Other interpersonal relationships. A rating of 2 or above would be regarded as a stressor.
Distress due to the tightening of social distancing measures^a^	Participants were asked to indicate their level of distress from experiencing the tightening of social distancing measures during fifth wave of COVID-19. A 11-point Likert scale (0 = Not stressed at all, 10 = Extremely stressed) was used.

Abbreviation: COVID-19 = Coronavirus disease 2019

^a^Tightening of social distancing measures refers to group gathering of more than 2 people in public place were prohibited, the maximum number of customers per table for catering premises were reduced to 2, dine-in ban after 6p.m. and closure of all recreational premises

### Statistical analysis

Network analysis was performed including network estimation and network comparison tests, derivation of centrality indices, and evaluation of network stability and accuracy. All variables were standardized by centring before inclusion in network analysis. All statistical analyses were conducted using R4.2.2.

#### Network estimation

A total of 12 variables were included in network analysis. Each variable was represented by a node, whereas the associations between variables were represented by edges. The model was estimated using the bootnet R-package. ‘EBICglasso’ default set was called for network modelling. This model selection estimated the network using graphical Least-Absolute-Shrinkage-and-Selection-Operator (LASSO) and selected the optimal model using Extended Bayesian Information Criteria (EBIC), with the hyperparameter set as 0.5 [29]. Graphical LASSO minimised false-positive associations by assigning penalties to shrink weak association to exact zero, resulting in a less dense network for easier interpretation [30]. Correlation coefficients, also known as edge-weights, ranged from − 1 to 1 were also generated from network analysis. They indicated both the strength and direction of association between two nodes after controlling for all other information. Network structure was plotted using the Fruchterman-Reingold algorithm from the qgraph R-package. Exploratory analyses were conducted to compare the overall network structure and global strength between genders, diagnosis (CMD vs. SMD) and history of contracting COVID-19 infection at fifth wave (yes vs. no). Comparisons were made using the NetworkComparisonTest R-package, a resampling-based permutation test that assesses differences between two networks [31].

#### Centrality indices

Four centrality indices were computed: Node strength quantifies how well a node is connected to others directly by summing the edge-weights connected to the node; Closeness measures the indirect connection a node has with others, reflected by the inverse of the sum of the shortest paths from a node to all other nodes; Betweenness refers to the number of times a node lies on the shortest paths between two other nodes; and Expected influence reflects the level of connectivity of a given node with other nodes in the network. All indices were calculated and visualized using the qgraph package in R. Predictability estimates were also computed for all nodes in the network. The estimates reflect how well each node is predicted by other nodes in the network [32] and are represented as pie charts around each node.

#### Network stability and accuracy

To examine network stability and accuracy, bootstrapping was applied to assess centrality and edge-weight parameters using the bootnet R-package. It was first applied to evaluate the stability of centrality indices by case-dropping. The indices were repeatedly calculated with different subsets of data that consisted of different proportions of data dropped. Stability was evaluated by the correlation stability coefficient (CS-coefficient), which is the maximum proportion of cases that could be dropped with a 95% certainty. A CS-coefficient above 0.5 indicates good stability [33]. Second, edge-weight accuracy was assessed by calculating their confidence intervals derived from 1000 non-parametric bootstrap samples. Third, bootstrapped differences tests were conducted to test for significant differences in edge-weights and node strengths using a set of 1000 bootstrapped sample.

## Results

### Characteristics of the sample

In our sample, 144 (34.7%) were male, the mean age was 40.6 years old (SD = 12.6), and 236 (56.9%) achieved secondary educational level or below. Two-hundred forty-six (59.3%) patients were diagnosed with CMD, and 169 (40.7%) with SMD. Detailed characteristics of the sample are summarized in Table 2.

**Table 2:** Characteristics of the study sample

	Mean (SD) / n (%)
*Demographics*	
Age, years	40.6 (12.6)
Gender (male)	144 (34.7)
Educational level	
Secondary or below	236 (56.9)
Tertiary or above	171 (41.2)
*Illness characteristics*	
Psychiatric diagnosis	
Common mental disorders	246 (59.3)
Severe mental disorders	169 (40.7)
Length of psychiatric service, years	8.3 (6.2)
*Psychopathological symptoms*	
Depressive symptoms (PHQ-9)	9.5 (7.4)
Anxiety symptoms (GAD-7)	7.6 (6.6)
PTSD-like symptoms (IES-R)	7.2 (6.0)
Insomnia symptoms (ISI)	11.7 (7.4)
Psychotic symptoms (CAPE-C15)	5.6 (2.2)
*Subjective cognition & quality of life*	
Subjective cognitive impairment	4.8 (4.2)
Health-related quality of life (SF-8)	25.4 (6.2)
*Psychological measures*	
Loneliness (UCLA-3)	4.9 (1.9)
Resilience (BRS)	2.9 (0.8)
*COVID-19 related factors*	
Fear of contagion	4.5 (3.2)
Number of COVID-19 related stressors	3.5 (2.8)
Distress due to social distancing measures	5.0 (3.3)

Abbreviations: BRS = Brief Resilience Scale; CAPE = Community Assessment of Psychic Experiences

Scale; COVID-19 = Coronavirus disease 2019; GAD-7 = Generalised Anxiety Disorder; IES-R = Impact of Event Scale– Revised; ISI = Insomnia Severity Index; PHQ-9 = Patient Health Questionnaire;

PTSD = Post-traumatic stress disorder; SF-8 = The Short Form-8; UCLA-3 = UCLA Loneliness Scale-3

### Network structure and analyses

**Fig. 1:**
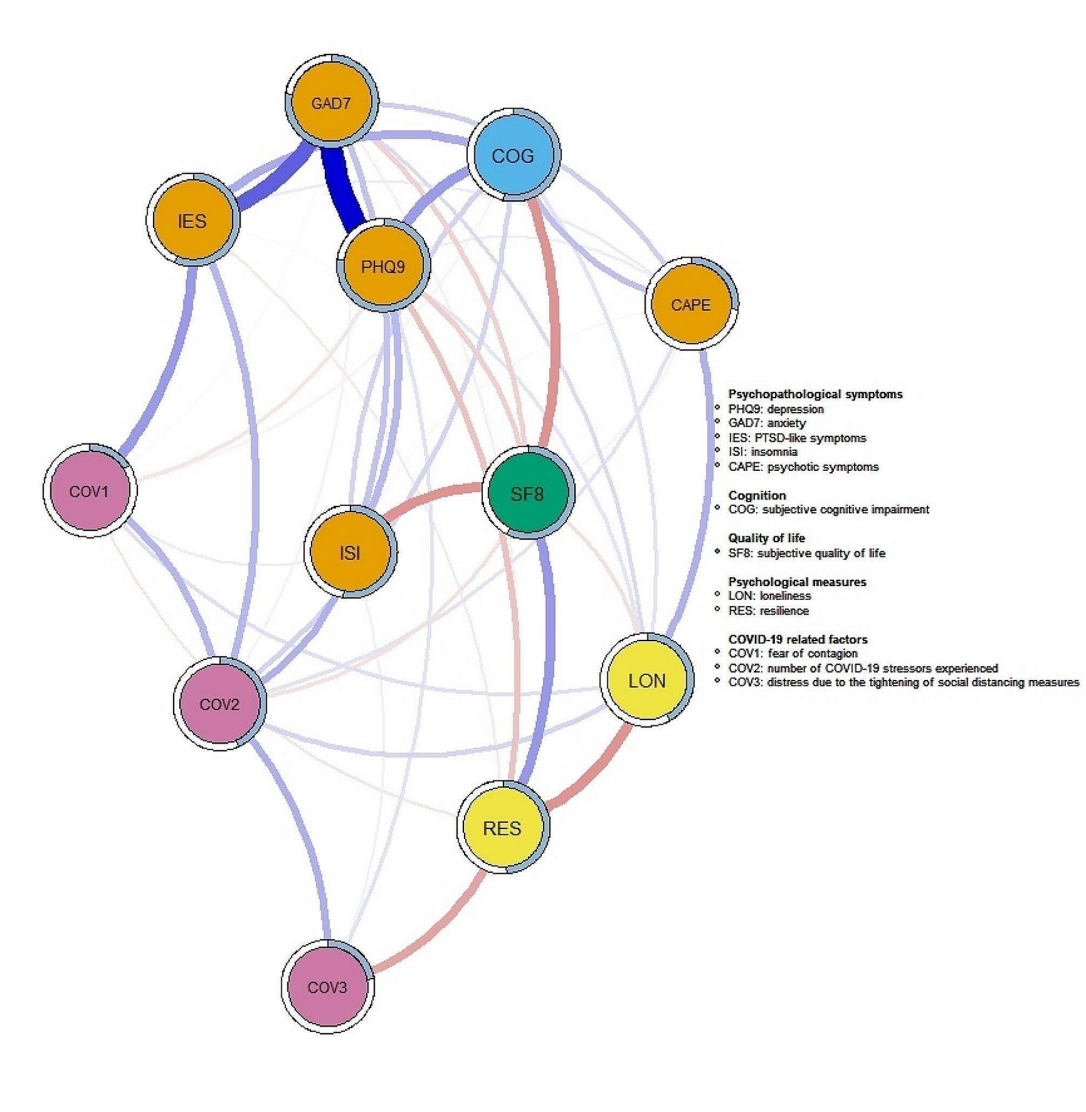
Network of psychopathological symptom, cognition, quality of life, psychological, and COVID-related variables. This is a network structure of 12 study variables. Each node represents a study variable and each edge represents a significant association between two nodes. Edge thickness reflects the magnitude of the association, in which thicker lines denote stronger associations. Blue lines indicate positive association while red lines denote negative association. Predictability estimates are represented by the circle around each node

The resulting network model illustrated all nodes were interconnected across domains (Fig. 1). Nodes from COVID-19 related factors and psychological measures were overall highly interconnected within domain, compared to nodes from other domains. Within psychopathological symptom domain, depression and anxiety were strongly associated (*r* = 0.52), followed by the association between anxiety and PTSD-like symptoms (*r* = 0.32), and the link between depression and insomnia (*r* = 0.14). Notably, interconnection across domains were observed in all psychopathological symptom nodes except anxiety. For example, depression was correlated to subjective cognitive impairment (*r* = 0.20), PTSD-like symptoms were associated with cognitive impairment (*r* = 0.17), fear of contagion (*r* = 0.20) and number of stressors experienced (*r* = 0.14) in COVID-19 related domain, while positive symptoms were connected with cognitive impairment (*r* = 0.13) and loneliness (*r* = 0.16). Quality-of-life also showed connections with several domains. It was negatively associated with insomnia (*r*=-0.22) and s cognitive impairment (*r*=-0.22), and was moderately correlated with resilience (*r* = 0.21). Lastly, number of COVID-19 stressors was associated with insomnia (*r* = 0.16), while distress due to social-distancing measures was negatively correlated to resilience (*r*=-0.18) (Fig. 1).

**Fig. 2:**
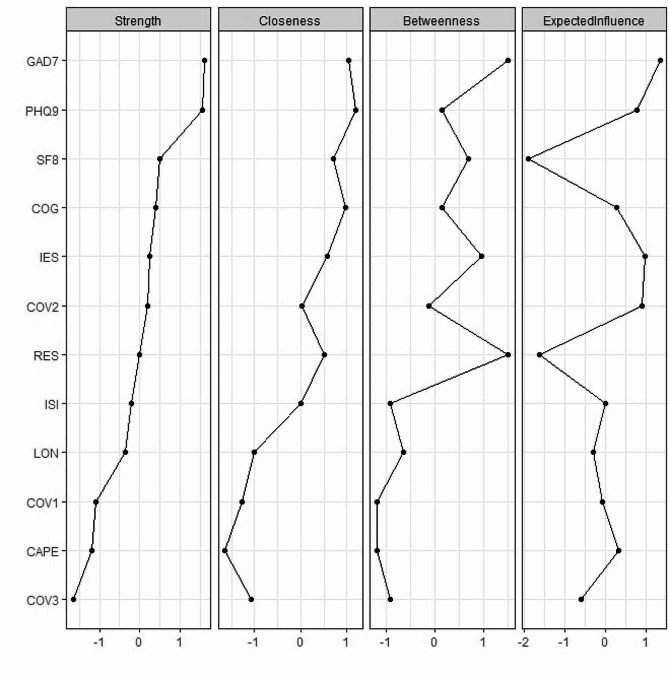
Centrality indices of study variables within the network. Centrality indices of node strength, closeness, betweenness and expected influence are shown as standard z-scores

As shown in Fig. 2, centrality analyses revealed that anxiety showed the highest node strength, followed by depression and quality-of-life. Anxiety also had the highest expected influence value, and ranked second for the degrees of closeness and betweenness among all variables in the network. In COVID-19 related domain, the number of COVID-19 stressors experienced demonstrated the highest centrality, with the highest node strength, closeness, betweenness and expected influence among three COVID-19 related variables. Depression and resilience showed the highest closeness and betweenness, respectively, among all variables in the network. For predictability, anxiety had the highest predictability (R^2^ = 0.78), followed by depression (R^2^ = 0.77). The mean predictability of the resulting network was 0.48 (SD = 0.19).

Network comparison between patients with versus without history of contracting COVID-19 revealed significant difference in structural invariance (*M* = 0.39, *p* = 0.002), but not in global strength (*S* = 0.78, *p* = 0.053). Specifically, the networks of infected and non-infected groups differed markedly in the association between fear of contagion and distress due to social-distancing measures (Supplementary Fig. S1). These two variables were negatively related to each other in the network of infected patients (*r*=-0.31), but were positively associated with each other in the network of non-infected counterparts (*r* = 0.07). Alternatively, network comparisons showed no significant differences between genders as well as between CMD and SMD patients in structural (gender: *M* = 0.25, *p* = 0.228; diagnosis: *M* = 0.18, *p* = 0.836) and global strength invariance (gender: *S* = 0.47, *p* = 0.215; diagnosis: *S* = 0.11, *p* = 0.767). The corresponding network per gender and diagnosis are depicted in Supplementary Figure S2 and S3, respectively.

### Network stability and accuracy

CS-coefficients for centrality indices were 0.75 for node strength and expected influence, 0.52 for closeness and 0.21 for betweenness. These indicated that the network had a good stability in node strength and closeness, whereas the index of betweenness should be interpreted with caution. Bootstrapped 95% CIs showed a narrow curve, suggesting reliable and accurate edge-weight estimates (Supplementary Fig. S4). Results from bootstrapped difference tests (Supplementary Fig. S5 and S6) revealed that most edge-weights and node strengths were statistically different from one another in the resulted network.

## Discussion

To our knowledge, the current study is the first to employ network analysis to examine complex inter-relationships among a comprehensive range of variables encompassing various psychopathological symptoms, cognitive complaints, loneliness and resilience, health-related quality-of-life and pandemic-related factors among psychiatric patients during COVID-19. Our results demonstrated that psychopathological symptoms and COVID-19 related factors were both highly connected within and between domains. Among the variables studied, anxiety played the most central role and had the strongest associations with other nodes. This indicates that emergence of anxiety symptoms in pandemic may lead to subsequent development of other psychopathological symptoms such as depression, PTSD-like symptoms and insomnia, with consequent deterioration of quality-of-life.

Three direct associations between the COVID-19 related factor domain and psychopathological symptom domain were observed from the constructed network. Number of COVID-19 stressors experienced was positively associated with PTSD-like symptoms and insomnia, whereas fear of contagion was solely linked to PTSD-like symptoms. Of these three connections, the last between-domain connection was the strongest. In line with COVID-19 literature on traumatic stress, our findings provided evidence that the pandemic itself can be inferred as a traumatic stressor [34]. For instance, fear of contagion could be regarded as an example of mind-wandering, in which individuals exhibit to cope with their distress but with deleterious effect on cognitive functioning [35]. Subsequently, frequent mind-wandering leads to poorer functioning in everyday life [36]. Alternatively, fear of contagion and economic hardship can constitute as subtypes of traumatic stress in COVID-19, which was found to predict PTSD, anxiety and depression in the general population [37]. Moreover, shortage of medical resources and health emergency are risk factors of developing PTSD-like symptoms during COVID-19 among healthcare professionals (HP) [38]. Although it could be argued that general population and psychiatric patients would be less likely to experience the same intensity of psychological distress compared to frontline HP at the COVID-19 outbreak, the aforementioned risk factors are still applicable to the former two groups. Indeed, studies conducted in HK at earlier pandemic stage indicated that worries of insufficient medical supplies and fear of contagion were associated with poorer mental-health outcomes [39, 40]. These suggest that COVID-19 may exert an indirect effect on individuals’ psychological distress by acting as continuous traumatic stressors in the fifth pandemic wave. Given their heightened susceptibility to stress, stronger associations between COVID-19 related factors and psychopathological symptoms may be more likely to emerge among psychiatric patients.

The COVID-19 stressor burden was also found to directly link with insomnia symptoms. Such relationship largely concurs with the literature on sleep research, including both conducted before and during the pandemic. According to the classic model of neuroendocrine stress response, exposure to stress activates the hypothalamic-pituitary-adrenal axis to release stress hormones, which subsequently modulate various physiological functions, including one’s sleep-wake cycle [41]. Prior studies have consistently reported higher prevalence of concomitant stress and insomnia symptoms experienced during the pandemic across different populations, such as general population, HP and psychiatric patients [42, 43]. Besides, a recent study demonstrated a longitudinal effect of increased stress on later perceived sleep disturbance during COVID-19 regardless of pre-existing sleep abnormalities [44]. This indicates a continuous effect of stress on individuals’ perceived sleep quality during the pandemic, in which sleep disturbance was positively associated with psychological distress [45]. Given the increased prevalence of sleep disturbance during pandemic [46] and its negative effect on quality-of-life, presentation of insomnia during pandemic should warrant attention and timely intervention is needed to minimize its adverse mental-health impacts.

Our exploratory analyses suggested network structural difference between psychiatric patients with versus without history of contracting COVID-19 during the fifth pandemic wave. Intriguingly, the resulting networks of infected and non-infected psychiatric patients differed markedly in the association between fear of contagion and distress due to social-distancing measures. These two variables were negatively related to each other among infected individuals but were positively associated with each other in non-infected counterparts. We speculate that such opposite directions of associations might partly be explained by potential differential psychological reaction toward COVID-19 between infected and non-infected individuals. A recent study indicated that individuals’ excessive fear reaction toward COVID-19 (or termed over-responses) was associated with heightened distress by self-isolation during the pandemic (e.g., social-distancing measures), whereas low fear reaction toward COVID-19 (or termed under-responses) was linked with increased tendency to disregard the social-distancing measures [47]. It might be possible that individuals with history of contracting COVID-19 (and recovered from the infection) perceived themselves as having low risk of infection, and hence exhibited under-responses toward COVID-19 with reduced fear of contagion compared to non-infected individuals. Consequently, participants in the infected group may experience greater distress due to social-distancing measures in the context of their diminished perceived threat against COVID-19. Nonetheless, owing to the scarcity of data examining network structural difference in relation to the history of COVID-19 infection, further investigation is required to verify our network comparison findings.

Anxiety symptoms and COVID-19 stressor burden play critical roles in leading to subsequent development of other psychopathological symptoms such as depression and insomnia. Future studies hence might consider to develop and evaluate effectiveness of interventions that target at reducing intensity of anxiety and stress level in different facets of daily life during pandemic so as to enhance the mental health and quality of life of psychiatric patients. For instance, a randomized controlled trial revealed that regular layperson-delivered telephone calls with empathetic conversational techniques could reduce anxiety, depression and loneliness, improving the general mental health during pandemic [48]). Another clinical trial showed that a single session of virtual reality with content to promote relaxation, distraction and stress relief demonstrated beneficial effect on reducing tiredness, shortness of breath, and anxiety, with an increase in the feeling of well-being during the pandemic [49]. Research like these ones can be extended to psychiatric patients to evaluate and maximize their clinical relevance.

There are several study limitations that warrant attention. First, the cross-sectional nature precludes us from investigating the change in psychopathological symptom severity among psychiatric patients prior to and during the pandemic. Second, causal relationships among variables could not be established. Third, our patient sample was recruited from outpatient clinics only and did not include those admitted to psychiatric inpatient units, and may therefore introduce selection bias towards patients with milder illness. Fourth, psychopathological symptom assessments were based on participants’ self-reporting (albeit well-validated and commonly used in mental-health surveys) which may not well align with the corresponding rating instruments administered by mental-health professionals.

In conclusion, this report is among the very few studies that comprehensively examined inter-relationships between psychopathological symptoms, cognitive complaints, health-related quality-of-life, loneliness, resilience and specific pandemic-related factors among psychiatric patients using network analysis. Our results indicate that anxiety is the most central node in the network, with a strong positive association with depression, and may represent a critical target of intervention to prevent further cascade of negative mental-health outcomes at times of pandemic. COVID-19 related variables including fear of contagion and stressor burden may exert influence on exacerbating psychopathological symptoms, especially insomnia and PTSD-like symptoms.

### Electronic supplementary material

Below is the link to the electronic supplementary material.

Supplementary Material 1

## Data Availability

The data that support the findings of this study and the analytic codes for data analysis are available from the corresponding author upon reasonable request.
