# Supplementary material for: Network analysis on psychopathological symptoms, psychological measures, quality of life and COVID-19 related factors in Chinese psychiatric patients in Hong Kong
Source: BMC Psychiatry. 2024 Apr 12;24:271. doi: 10.1186/s12888-024-05690-7 (PMC11010282; doi:10.1186/s12888-024-05690-7)
Supplement: Supplementary file 1 — Supplementary Material 1 [file 12888_2024_5690_MOESM1_ESM.docx]

**Supplementary Materials**

1. **Figure S1**. Networks of psychopathological symptom, cognition, quality of life, psychological, and COVID-related variables for psychiatric patients contracted COVID-19 infection (Left) and those without history of COVID-19 infection (Right) during the fifth pandemic wave.
2. **Figure S2**. Networks of psychopathological symptom, cognition, quality of life, psychological, and COVID-related variables for female (Left) and male (Right) psychiatric patients.
3. **Figure S3**. Networks of psychopathological symptom, cognition, quality of life, psychological, and COVID-related variables for patients with common mental disorders (Left) and those with severe mental disorders (Right).
4. **Figure S4**. Bootstrapped 95% confidence intervals of edge-weights for the estimated network.
5. **Figure S5**. Bootstrapped difference tests between non-zero edge-weights in the estimated network.
6. **Figure S6**. Bootstrapped difference tests for node strength of the estimated network.


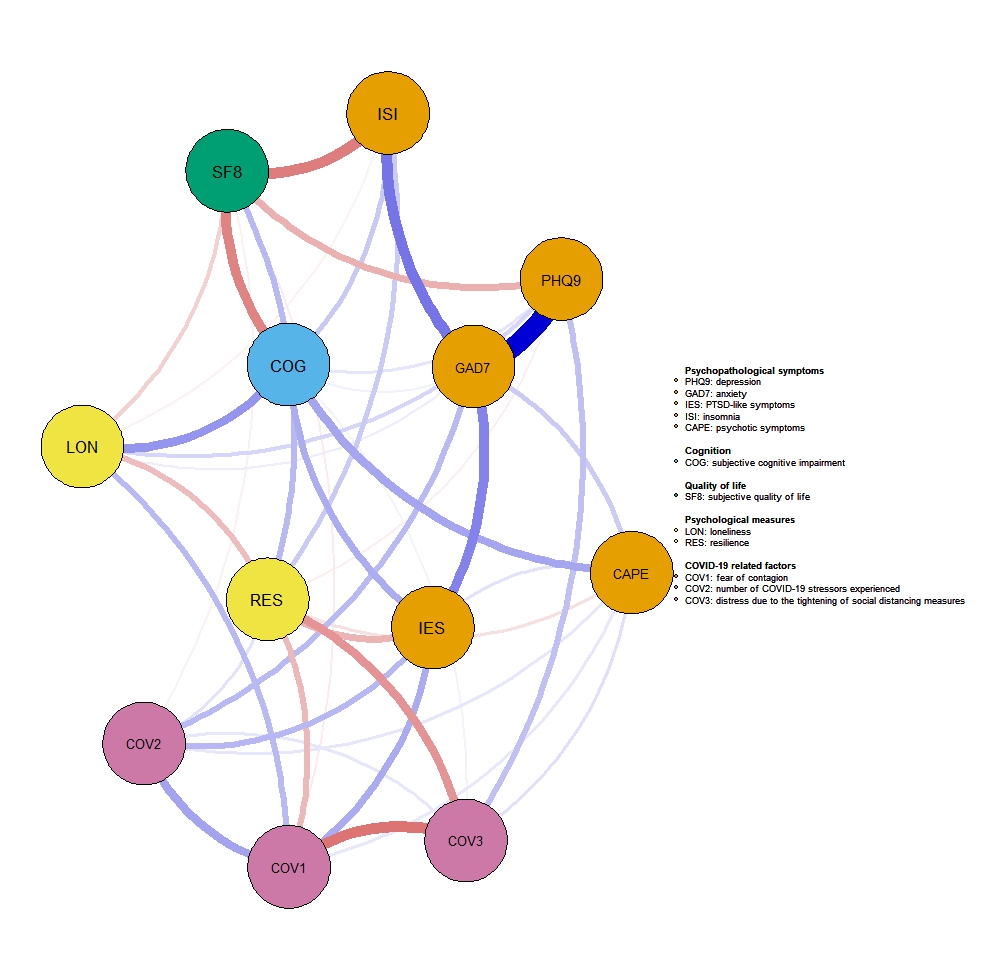

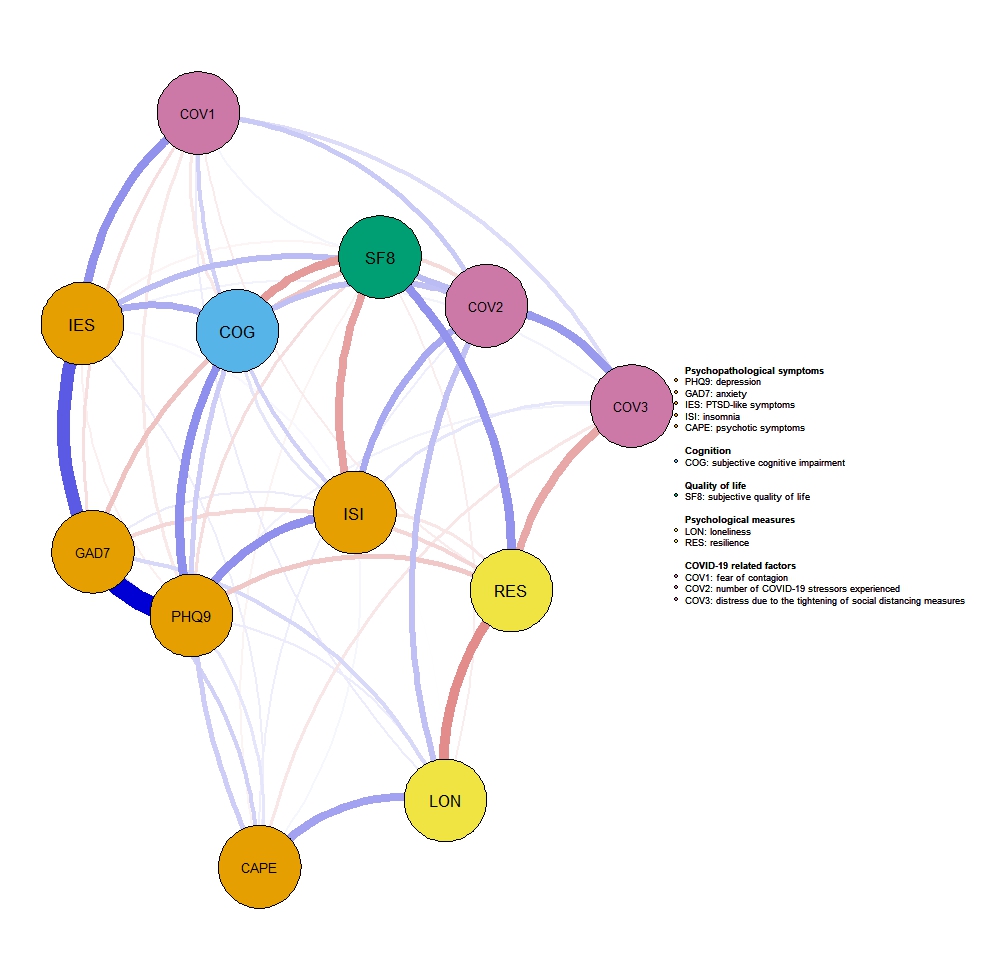


**Fig. S1.** Networks of psychopathological symptom, cognition, quality of life, psychological, and COVID-related variables for psychiatric patients contracted COVID-19

infection (Left) and those without history of COVID-19 infection (Right) during the fifth pandemic wave.


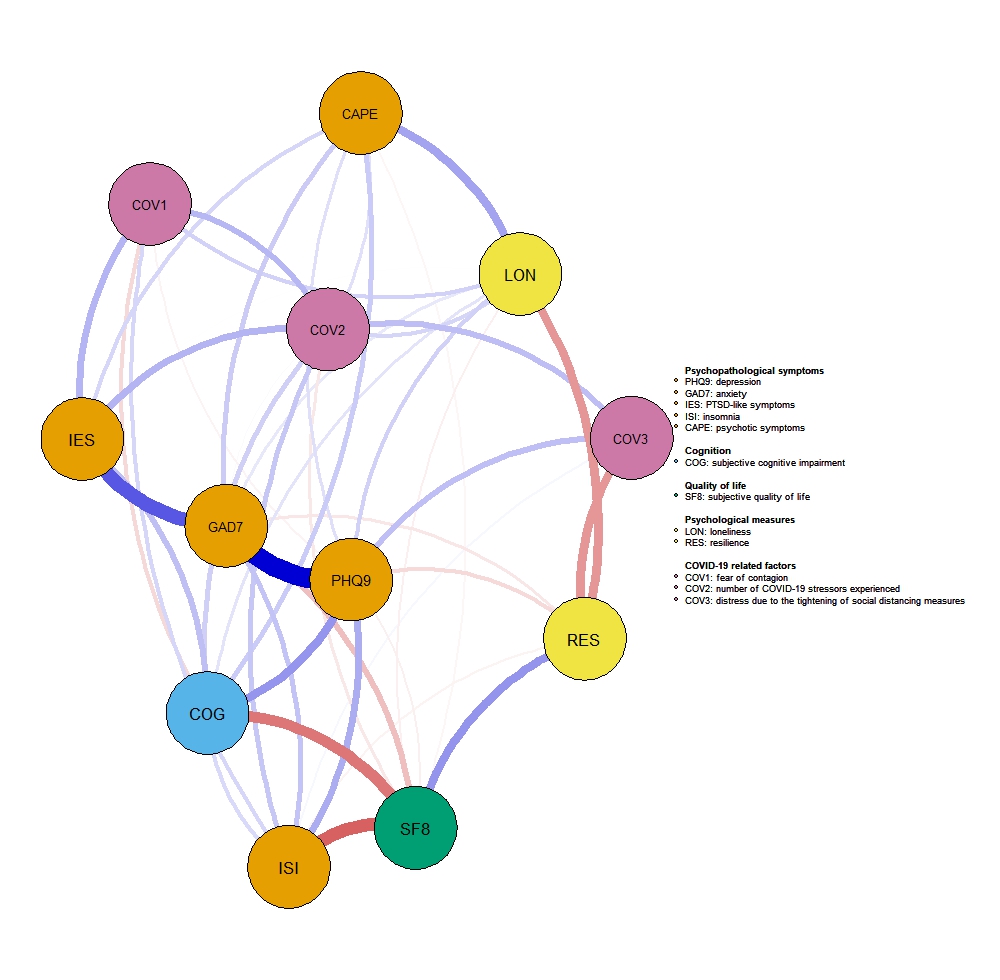

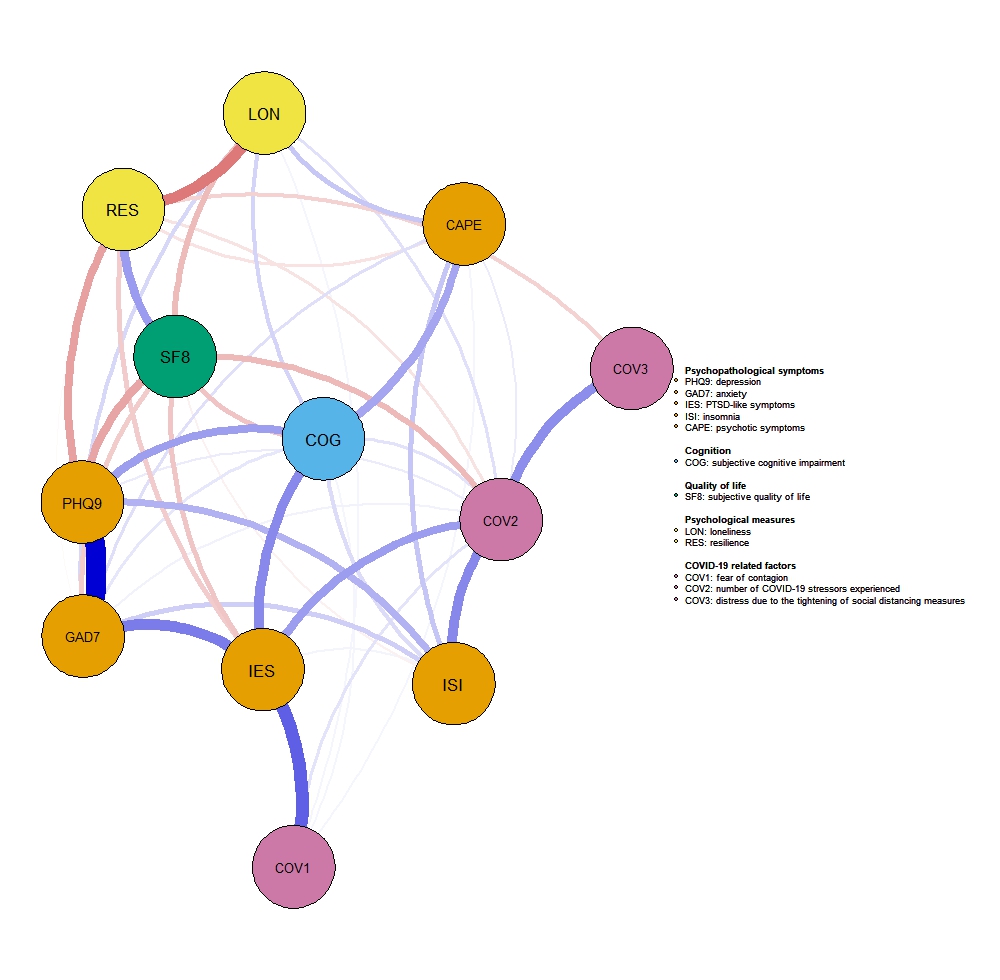


**Fig. S2.** Networks of psychopathological symptom, cognition, quality of life, psychological, and COVID-related variables for female (Left) and male (Right) psychiatric patients


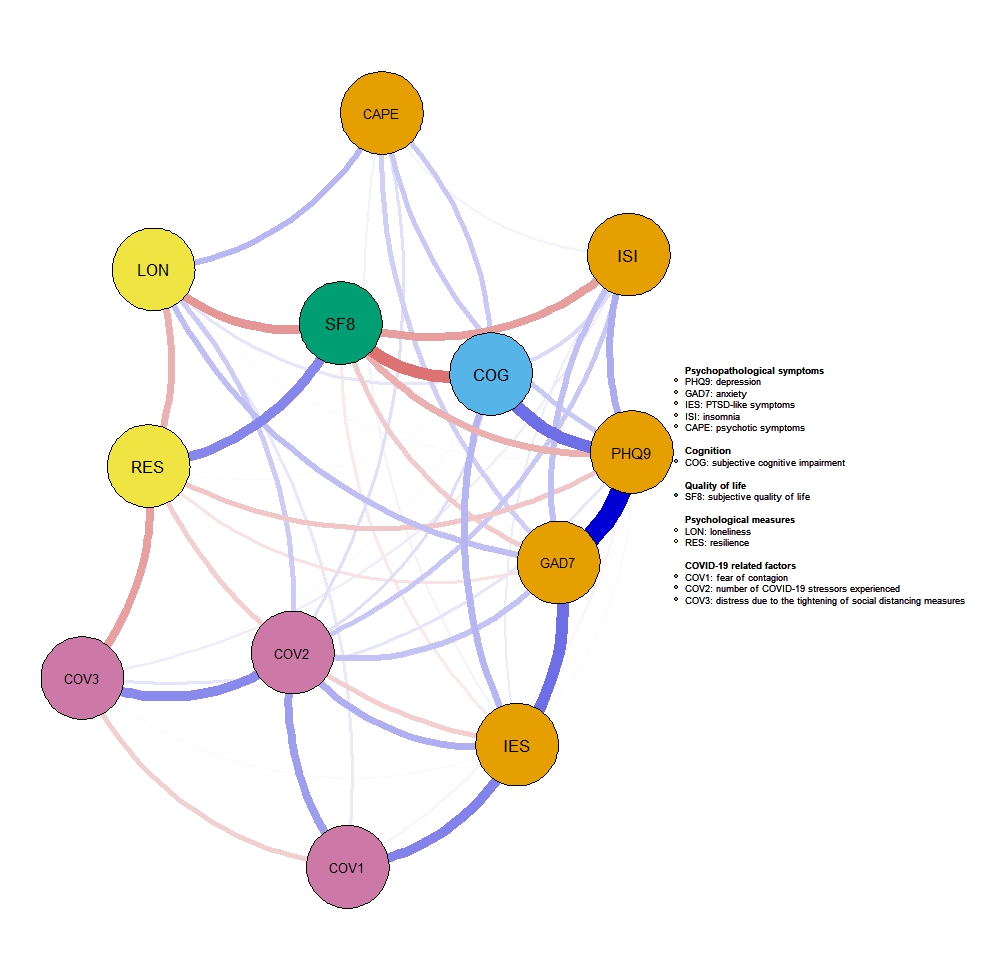

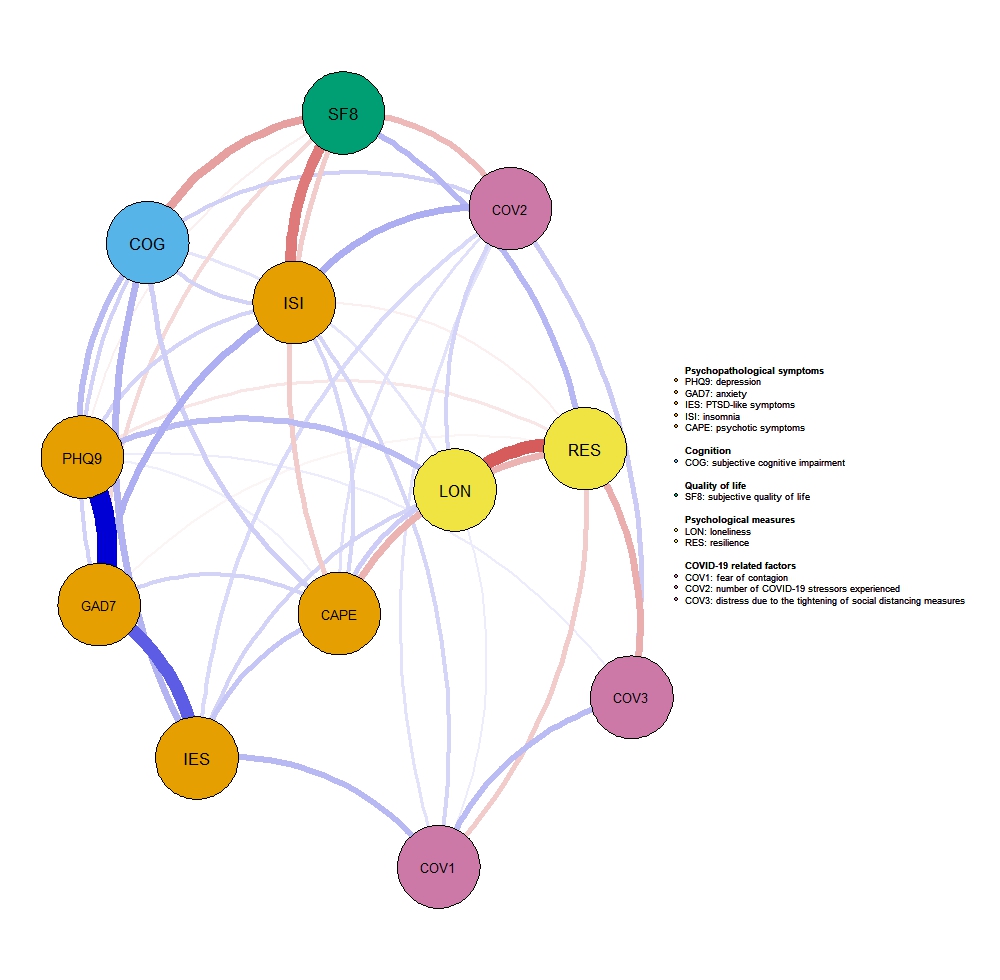


**Fig. S3.** Networks of psychopathological symptom, cognition, quality of life, psychological, and COVID-related variables for patients with common mental disorders (Left)

and those with severe mental disorders (Right).

**
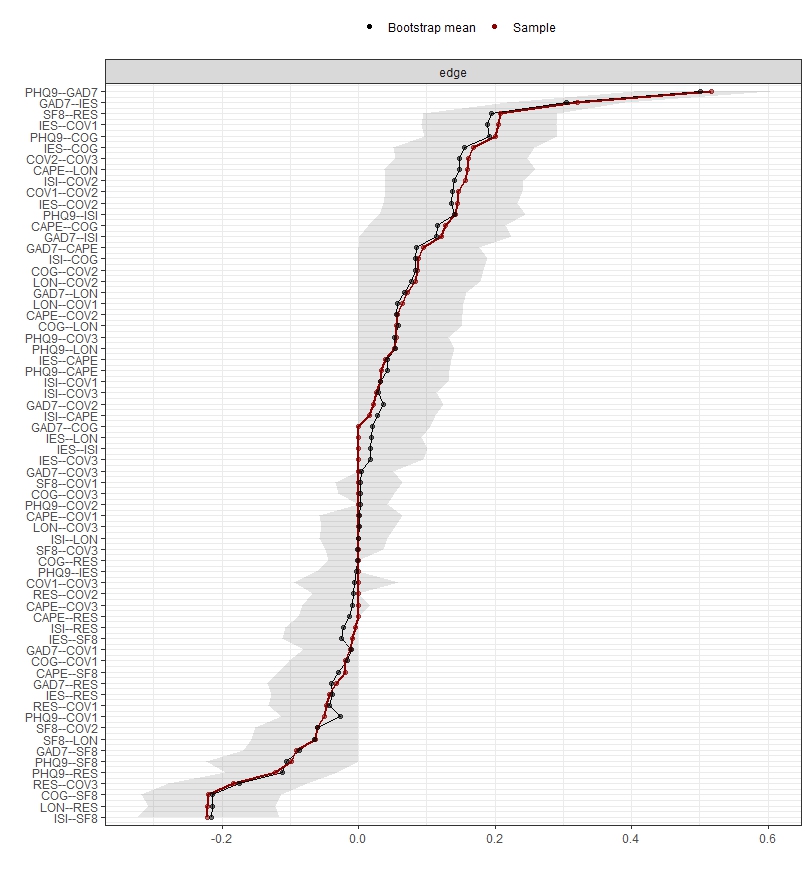
**

**Fig. S4.** Bootstrapped 95% confidence intervals of edge-weights for the estimated network.

The red line indicates the sample edge-weight (sorted in increasing order in x-axis) and the gray

area is the bootstrapped confidence intervals.


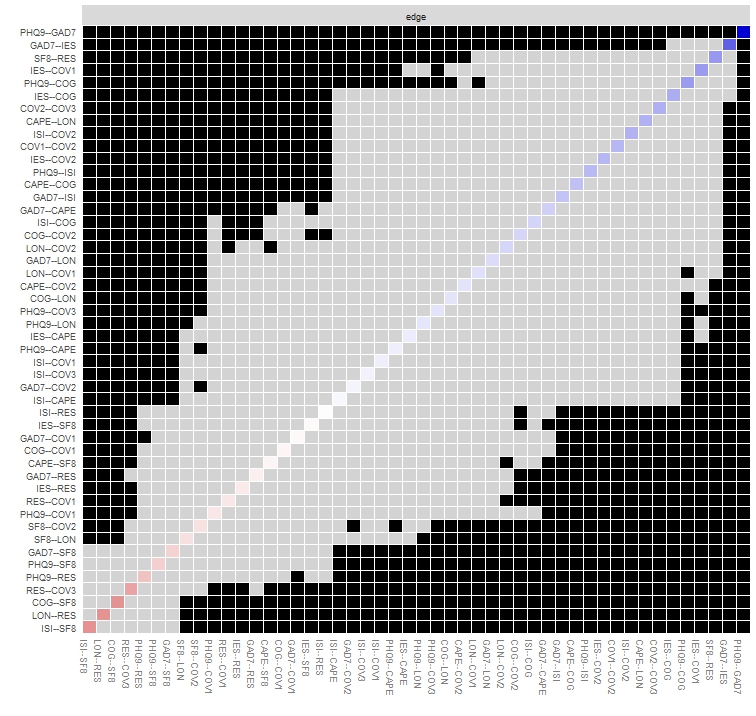


**Fig. S5.** Bootstrapped difference tests between non-zero edge-weights in the estimated network.

Gray boxes indicate edge-weights that do not differ significantly from one another, while black boxes indicate edge-weights that do differ significantly. Blue and red boxes on the diagonal correspond to edge-weights with positive and negative correlations, respectively.


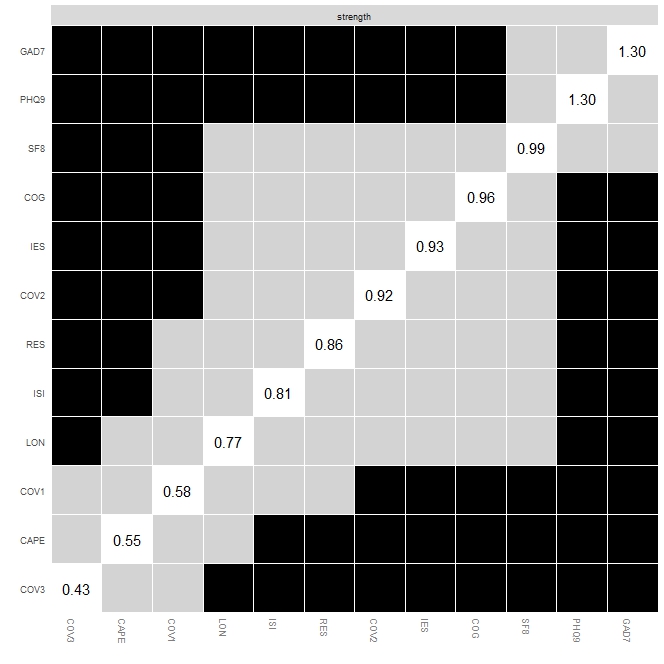


**Fig. S6.** Bootstrapped difference tests for node strength of the estimated network.

Gray boxes indicate node strengths that do not differ significantly from one another, while black boxes indicate node strengths that do differ significantly. The number in the white boxes (i.e., diagonal line) represent the value of node strength.
